# Education in Vaccinology: An Important Tool for Strengthening Global Health

**DOI:** 10.3389/fimmu.2018.01134

**Published:** 2018-05-24

**Authors:** Paul-Henri Lambert, Audino Podda

**Affiliations:** ^1^University of Geneva, Geneva, Switzerland; ^2^GSK Vaccines Institute for Global Health, Siena, Italy

**Keywords:** training, vaccinology, education, global health, vaccines

## Abstract

Over the past 20 years, education of scientists and public health professionals in Vaccinology has increased dramatically. There are now many international, regional, and national courses that provide education in vaccinology. The proliferation of these courses and the high number of applications submitted demonstrate the increasing and continuous need for improved education in this field since, generally, comprehensive vaccinology training is not offered to medical and/or biological sciences students as part of their Universities courses and consequently there is insufficient knowledge of vaccine topics among health-care providers. Multidisciplinary vaccinology courses have not only educational purposes but they may also contribute to strengthening the development, testing, and use of vaccines, which remain the most efficient tool for infectious disease prevention. The courses available have a varied focus and prioritize topics based on the trainees’ different levels of professional exposure and requirements. Overall, they might be classified in two key categories: (i) courses targeting students who, after their university studies in Medicine, Biology, etc., develop a strong interest in vaccines, would like to learn more about the various aspects of vaccinology, and potentially develop a career in this field (postgraduate courses); (ii) courses targeting postdoctoral professionals, who already have a sufficiently broad knowledge of vaccinology, but would like to develop stronger skills to be able to play a leading role in decision-making for vaccine development (advanced professional courses). Both postgraduate and professional courses are available and are based on comprehensive curricula. In the future, particular attention should be paid to include in the training curricula topics that might help vaccine development, efficient and sustainable vaccine introduction through epidemiologically sound vaccination programs, and best practices to address associated challenges, including vaccine hesitancy which could become a threat to successful implementation of vaccination programs, particularly in developed countries. In addition, it appears that the next phase of vaccinology training could benefit from a global and more structured platform that could facilitate exchanges and collaboration and amplify the current capacity for disseminating vaccine education for future vaccinology leaders around the world. This would be favored by synergizing the efforts currently devoted to vaccinology education. To initiate this process of analysis and systematization, a multinational effort is needed.

## Introduction

Education in vaccinology is an important priority to strengthen development, testing and use of vaccines, which remain the most efficient tool for the prevention of infectious diseases both in developed and developing countries. The several courses available worldwide today have a different focus and curricula are tailored to the trainees’ different levels of professional exposure and requirements (1, 2). Overall, they might be classified in two key categories: (i) courses for more junior scientists who, after completion of their biological studies at University, would like to know more about vaccines and vaccinology and might potentially develop a career in this field (postgraduate courses); (ii) courses for experienced scientists who already have a quite good knowledge of vaccinology and are ready to develop a deeper competence to lead vaccine development projects at various levels of responsibility and to actively participate in strategic groups deciding on vaccination policies at national, regional, or international levels (advanced professional courses).

## Postgraduate Courses in Vaccinology

Some of the disciplines representing the fundamental scientific background for efficiently working in a vaccine development environment, such as clinical aspects of infectious diseases, microbiology, immunology, epidemiology, biostatistics, and others, are regularly taught in University courses; however, most often these courses do not have a focus on the whole vaccine development process or on the public health context for the introduction of new vaccines and rarely are these disciplines presented with a multidisciplinary and holistic approach (3). In addition, theoretical teaching is not enough, and there is also a need for practice-based training and exposure to vaccine development-orientated activities. This is particularly true for disciplines that are not usually taught in university courses, such as Pharmacovigilance, Regulations, and Ethics in vaccine R&D studies and, particularly, aspects related to animal and human research. In this regard, internships within an experienced project team are an opportunity not only to allow young scientists to learn day-by-day vaccine development work but also to introduce them into the dynamics of a scientific community working together toward a common goal.

Multidisciplinary vaccinology courses are an important priority particularly for scientists from developing countries where vaccines have significantly contributed to the dramatic decrease in the number of deaths, due to infectious diseases, particularly in children below 5 years (4). However, in these countries, almost five million children still die every year and many of these deaths are due to vaccine preventable diseases. Therefore, there is a huge need not only for new vaccines against diseases mostly affecting developing countries, for which a vaccine is not yet available, but also for significant efforts and resources to introduce in Africa, Asia, and in general in low- and middle-income countries (LMIC) vaccines that are already available to children of developed nations. Development and introduction of new vaccines in these countries is obviously dependent on availability of locally generated data, particularly the high-quality clinical data needed by regulatory authorities for vaccine registration and by WHO for vaccine pre-qualification. An essential requirement to make this happen is to have a cohort of well-trained scientists from developing countries who have a clear understanding of the whole process behind vaccine development and subsequent vaccine distribution. With these capabilities, local scientists may become active players and efficiently implement the various activities needed for registration of new vaccines and then support post-licensure vaccine introduction in the context of country tailored immunization campaigns. Therefore, vaccinology courses for scientists from developing countries should include classes on epidemiology and clinical development, but also education on public health systems operations, cold chain logistics, and vaccine distribution. Given the challenges associated with such extensive vaccinology training, identification of suitable candidates for the training activities is really key. Participants may have different educational backgrounds and different R&D experience; therefore, well thought selection criteria based on a grading system should be established upfront to make sure that selected candidates can get the most from the training activities.

An example of this approach is given by the Master in Vaccinology and Pharmaceutical Clinical Development of the University of Siena (5), which one of us, AP, contributed to set up and implement. This course, a collaborative effort between academia and vaccine industry, particularly tailored for young physicians from developing countries, is an 18-month program, combining theoretical and practical training. The theoretical teaching component includes 10 modules in the key vaccinology disciplines (Public Health and Vaccine Development Process; Immunology and Preclinical Research; Manufacturing and Quality Control Processes; Infectious Diseases and Vaccine Prevention; Clinical Development Methodology, Biostatistics and Clinical Data Management; Pharmacovigilance; Epidemiology, Health Systems and Economics; Good Clinical Practices, Clinical Quality Assurance and Clinical Trial Operations; Regulatory Affairs; Policies and Recommendations for Vaccines in the World) and, in addition, parallel educational seminars for personal and professional development. This extensive theoretical training is supplemented by a 7-month training, at the University of Siena and within different departments of the sponsors and collaborative institutions, followed by investigational site training. Finally, the value of this course to the students is maximized by a faculty including worldwide experts from well-known international universities, supranational organizations, and vaccine industry (5).

## Advanced Courses in Vaccinology

In the past 20 years, there was an explosive development and introduction of new vaccines that have or may have a considerable impact on public health strategies. As a result, there is now an increasing need for experts with a broad understanding of major issues in vaccinology. This need exists as well in industry, including major players and subject matter experts, as in academia and public health. In fact, it is of critical importance for decision makers in industry to understand the needs and the determinants that will influence the use of a given new vaccine in various country settings. Similarly, experts involved in public health strategy and in decisions related to the introduction of a new vaccination program at national, regional, or international levels must know key issues in the development process, essential safety considerations, limitations of the manufacturing process, and vaccine-related economic issues, e.g., cost-effectiveness. Managing real or alleged post-licensure safety issues is of critical importance. These aspects are also of great concern for academic professionals involved in training scientists with a potential role in vaccine development or monitoring.

**Figure 1:**
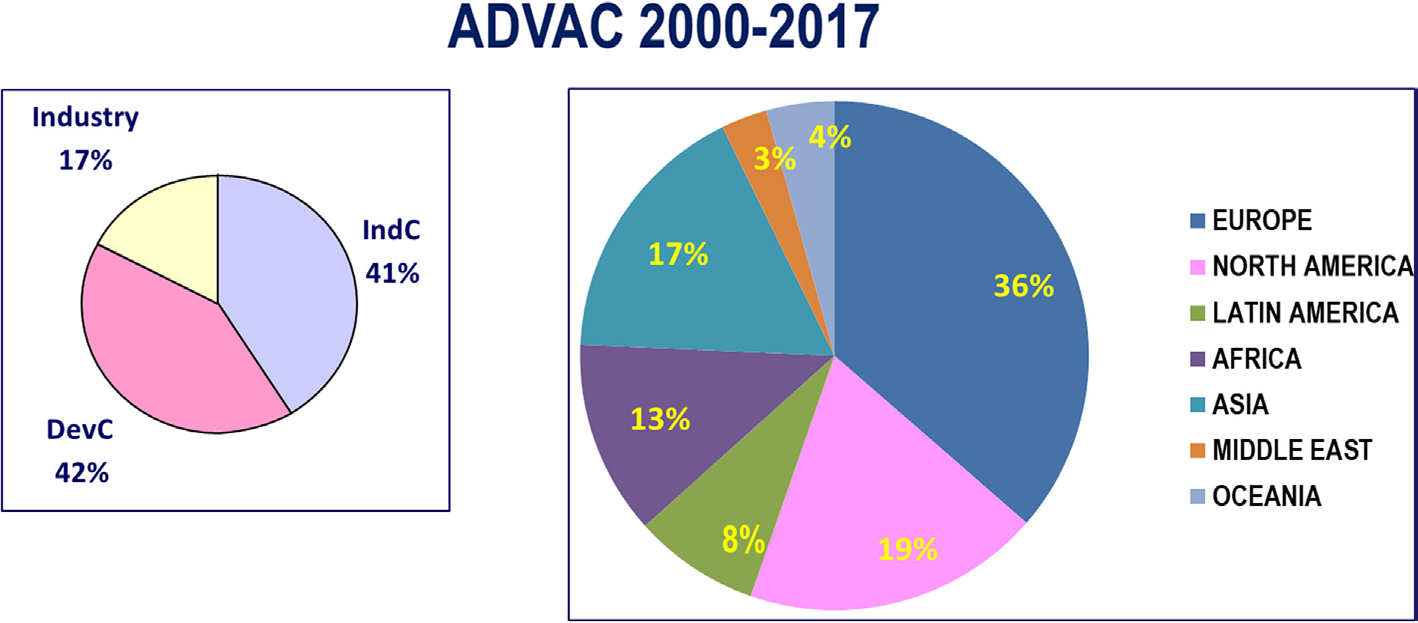
Geographic distribution of ADVAC participants from 2000 to 2017.

A good example of this type of training is the Advanced Course of Vaccinology, ADVAC, which one of us, PHL, contributed to set up and implement. This course, organized on an annual basis since 2000 by University of Geneva and Fondation Mérieux, at Veyrier-du-Lac (France), in partnership with WHO, Johns Hopkins SPH & CDC and support from the European Commission and the Bill and Melinda Gates Foundation (6). At inception, it was aiming at filling major gaps in global vaccination strategies: (i) a lack of scientists with a broad vision of issues related to vaccines and immunization, (ii) a lack of qualified decision makers to identify priority targets in vaccinology, and (iii) a lack of qualified policy makers for deciding on the introduction of new vaccines in vaccination programs. Since 2000, 18 courses have been organized, gathering in total 1,070 participants from over 100 countries (Figure 1). To ensure a maximal impact, it appeared of particular importance to select highly motivated candidates, likely to have soon increasing responsibilities. It was also critical for appropriate networking to maintain a course format allowing the mixing of people with diverse professional backgrounds and diverse geographic origin: 41% came from high-income countries, 42% from LMIC, and 17% from industry (Figure 1). The ADVAC curriculum is providing a broad view of the various aspects of vaccinology: (1) priority targets for vaccine R&D, (2) understanding vaccine-induced immune responses, (3) new vaccine approaches, (4) clinical assessment of vaccine efficacy, (5) vaccine safety and regulatory aspects, (6) decision-making process for introduction of new vaccines, (7) defining optimal vaccination strategies, and (8) dealing with real or alleged adverse effects. The success of these courses is certainly dependent on the quality of the lecturers who are all top level vaccinologists on the international scene. However, a key factor is the interactive nature of all sessions, particularly in small groups or during group exercises including role play sessions and informal debates. The concurrent evaluation of training sessions is particularly helpful to adjust the level of training to the needs of the students. A follow-up program for ADVAC alumni has proven effective to maintain and increase the network of vaccinologists that is resulting from the initial training effort.

## Discussion

Several postgraduate and advanced professional courses are available for training of junior and senior scientists, interested to deepen their respective knowledge in vaccinology. As shown by the examples mentioned in this review, some of these courses have already good multidisciplinary curricula; however, looking at the challenges and gaps that still limit the expansion and the sustainability of vaccination programs, there are a number of topics that should be more deeply addressed in future trainings.

An important gap toward expansion of vaccination in developing countries, particularly in Africa, is the lack of a sufficient manufacturing capacity that could enable local development and production of new vaccines, thus making vaccination programs sustainable in most of LMIC once GAVI support is over. Among other factors, development of local manufacturing capacity is affected by lack of a well-trained and competent pool of local scientists and technicians who could reliably support technical operations ranging from technology transfer activities to development, formulation, manufacturing, quality control, and release of vaccines.

Recently, some training courses on these aspects have been organized by WHO, also in collaboration with both public and private institutions (7, 8). In addition, as part of the ADITEC project funded by the European Commission (9), the WHO and the University of Lausanne organized various theoretical and practical 1-week courses in “Adjuvants and vaccine formulations” with the objective of training students on production, purification, characterization, and control of recombinant antigens, and on methods of preparation of adjuvants, including oil-in-water emulsions and aluminum gels, their formulation with antigens, and quality control of the resulting vaccines. Outcome of these technical trainings was excellent, based on the feedback received, and similar initiatives should be more frequently organized and offered to fruition in the future.

Anti-vaccination sentiments are heterogeneous beliefs, commonly defined as vaccine hesitancy, may represent an important cause of reduced vaccination coverage, both in developing and in developed countries, and sometimes may lead to recrudescence of infectious diseases for which vaccines have been available for a long time (10). Despite their unquestionable contribution to the reduction of morbidity and mortality from infectious diseases and, more in general, to an increased level of public health worldwide, for several reasons, mostly unfounded, vaccines have been associated to negative perceptions about their safety and, consequently, a growing sense of mistrust is associated with their use and should be properly addressed. Adequate education of health-care professionals is of paramount importance to address and reduce parental anxiety, concerns, and fears and therefore vaccinology trainings should more and more include well-documented sessions on vaccine safety. Similarly important is that vaccinologists are appropriately educated also on the potential side effects of vaccination, including identification and quantification of risks, so that, providing balanced and respectful information, they may contribute to re-establishment of trust (11).

An alarmingly high number of emerging bacterial infections are caused by the increasing anti-microbial resistance (AMR) worldwide and they may play an even worse effect on global morbidity and mortality in the near future (12). This is largely due to excessive and often inappropriate use of new antibiotics in medical practice and to the poorly controlled antibiotic use in animal food industry. Education of vaccinology scientists on the achieved reduction of antibiotics use and AMR by vaccination, with tangible benefits going beyond the non-vaccinated populations, through herd immunity, might push toward development of new future vaccines having also AMR reduction in their target product profile. This might also lead, on the one side, to better quantify the magnitude of antimicrobial use and of AMR for a given vaccine preventable disease and, on the other side, to select more appropriate vaccine candidates, including vaccines against highly resistant serotypes of the pathogen and/or virulence factors relevant for resistance acquisition.

Some other aspects deserve more and more attention in future vaccinology trainings; they include (i) preclinical and clinical vaccine assessment in LMIC, (ii) financing of vaccination programs, (iii) vaccine delivery, (iv) vaccine introduction strategies, and (v) vaccine regulations.

## Conclusion

Our vision of the future of vaccinology, and associated medical and social impacts, is that more and more scientists will be required for the implementation of all aspects of the vaccinology lifecycle process, from vaccine research to optimal vaccine use in the field. Therefore, the importance of appropriately developing the technical skills of next generation vaccinologists is paramount, best initiatives currently devoted to vaccinology education should join forces and, with a multinational effort, a global and structured platform for future training of vaccine scientists around the world should be developed. To achieve this goal, a global commitment to provide continuous education and training is needed from all stakeholders, including Academia, Industry, and Public Health Institutions, with the ultimate objective of ensuring sustainability of life saving vaccination programs at the global level.

## Author Contributions

Both authors contributed equally to this manuscript.

## Conflict of Interest Statement

AP is an employee of the GSK group of companies and is actively involved in several vaccine development programs and vaccinology education initiatives. P-HL has no potential conflicts to disclose. The reviewer AP declared a shared affiliation, though no other collaboration, with one of the authors AP to the handling Editor.
